# Improving quality outcomes via process improvements and innovation: the largest single-surgeon series of 1,701 consecutive robotic lobectomy and segmentectomy cases

**DOI:** 10.3389/fsurg.2025.1589149

**Published:** 2025-04-24

**Authors:** Robert J. Cerfolio, Natalie A. Ostro, Ashley J. McCormack

**Affiliations:** Department of Cardiothoracic Surgery, New York University Langone Health, New York, NY, United States

**Keywords:** robotic, lobectomy, segmentectomy, quality improvement, process implementation and change, robotic surgery

## Abstract

**Objectives:**

Our goal is to continuously improve patient outcomes, care quality, and overall experience.

**Methods:**

This is a quality improvement study based on the experience of a single surgeon and represents the world's largest reported consecutive series of robotic lobectomy and segmentectomy performed by a single surgeon.

**Results:**

From 1 January 2009 to 31 December 2024, a total of 1,701 patients (52% women) were treated, of whom 1,138 underwent robotic lobectomy (1,094, 96.1% were completed robotically) and 563 patients underwent segmentectomy (561, 99.6% were completed robotically). Quality metrics improved over each quartile: conversion rates decreased from 13 patients in our first 62 operations to 1 in our last 600 patients (*p* < 0.001), 90-day mortality decreased from 0.3% to 0% (*p* < 0.001), and major morbidity decreased from 6% to 1% (*p* < 0.001). Among patients with cancer, 99% underwent an R0 resection, with a median of five N2 and two N1 lymph node stations resected, 24 lymph nodes removed, and blood loss of 20 cc. Efficiency metrics improved with medians as follows: length of stay decreased from 110  to 26 h (*p* < 0.001), operative times fell from 125 to 93 min (*p* < 0.001), chest tube duration decreased from 72  to 4 h, and patient satisfaction scores improved from 87% to 98%. Various selective process improvements and strategies that we implemented and, in our opinion, improved both patient outcomes and experience are shared to scale this experience to others.

**Conclusions:**

A commitment to getting better via innovation and process improvements of all aspects of the pre-, intra-, and postoperative care and their pathways leads to improved outcomes and patient experience for robotic pulmonary resection. The selective processes and strategies that we believe led to these improving outcomes are shared and are possibly scalable elsewhere.

## Introduction

Pulmonary lobectomy and segmentectomy have progressed significantly over the last 25 years. In the past, thoracic surgeons routinely used thoracotomy, epidurals, arterial lines, urinary catheters, type and cross-matching, and postoperative intensive care. Patients typically had a 5–7-day length of stay, with a 2% 30-day and a 3%–4% 90-day mortality (1, 2). Ten years later, our approach has evolved to a minimally invasive platform (we favor a robotic one) which we use in nearly all patients. We typically place one or two peripheral intravenous lines only, perform an efficient 1.5 h minimally invasive robotic operation, have almost no conversions to thoracotomy, require a 23 h length of stay, and remove chest tubes before patients leave the operating room or within a few hours in the recovery room. As a result, patients enjoy essentially no major morbidity or 90-day mortality (3). Most can return to work in a few days or some in a week or two. In addition, the survival rates have also improved (3). These improved outcomes have been forged by the innovations of many physicians around the globe in which every part of our process has been dissected, studied, improved, and shortened all to improve our collective patients' outcomes and experience. We too have used a team approach and leveraged our expert pulmonologists, anesthesiologists, and thoracic surgeons and based many of our process changes on not just data but also on vision that does not yet have a *p*-value or prospective randomized trial data.

The goal of this manuscript is to review some of the process improvements that we have implemented in our practice over the 15-year span of this study that we believe have allowed us to improve our outcomes and patient experience. Most importantly, we aim to share the lessons we have learned that we believe others can scale in their institutions understanding the difference in culture and resources. Neither we nor others can prove the cause and effect of the processes we choose to report as the direct cause of the improvement. We cannot know that one change or process improvement was the sole reason for the overall improved outcome or patient experience. Undoubtedly, time itself is a non-controllable variable since most all of us develop better patient selection and, by definition, gain more experience each day in the operating room. We offer our perspective and our experience and identify the processes we believe were critical in our improvement. Finally, there remain many other opportunities for us to further improve to continue to get better and improve the care and experience that all of our patients receive.

## Patients and methods

### Study design

This is a quality improvement initiative led by one surgeon (RC). All perioperative data were collected, reviewed, and contained in a database. The study design, including a waiver of patient consent, was approved by the Institutional Review Board at NYU Langone Health #s23-01042. The primary outcomes were quality metrics including conversion rates, operative blood loss, 30- and 90-day mortality rates, and major morbidity. In addition, we tracked and reported efficiency metrics including median length of stay, median operative times, and chest tube duration. Operative time was defined as skin incision to skin closure time. The secondary outcome was patient satisfaction as measured via the Hospital Consumer Assessment of Healthcare Providers and Systems (HCAHPS) scores from nationally standardized and publicly reported surveys. This was the sole source used to assess patient satisfaction scores.

### Perioperative management

All patients were evaluated in a standard fashion for lung resection using testing such as computed tomography scan, integrated positron emission tomography, and pulmonary function testing and stress test in selected patients as we have previously published (4). Lung resection was conducted with a da Vinci Xi surgical system (Intuitive Surgical, Sunnyvale, CA, USA) through a portal four-arm approach and an additional assist port, as we previously reported (5), with minor changes such as placing the robotic ports above the ninth rib and the most posterior port 4 cm distal from the spinous process.

At the conclusion of the operation, a single 20-French chest tube is inserted in the access port, positioned apically and posteriorly, and then connected to a digital drainage system, previously Thopaz (Medela Healthcare, Baar, Switzerland, used from 2018 to 202) and now Thoraguard (Centese, Omaha, NE, USA). We ensure the lung fully inflates visually with the camera at the end of the operation. From 1 March 2023, we have attempted to remove the tube prior to the patient leaving the operating room and close the tube site with a subcuticular suture. Four years prior, we removed the chest tube in the recovery room within 4–12 h as published (6, 7). A brief summary of tube management then is as follows: patients are given ice cream in the recovery room and chest tubes are removed if (1) the patient is clinically stable without low pulse oximetry reading for that patient's baseline; (2) there is no new or enlarging subcutaneous emphysema; (3) the chest X-ray (CXR) shows either complete lung expansion or fixed pleural space deficit (8); (4) there is no cloudy, milky, or frankly bloody chest tube effluent; and (5) there is no air leak. If there is an air leak, then the chest tube is left in place. If there is a cloudy effluent that is suspicious for chyle, the chest tube is kept in place, and the drainage is sent for a triglyceride level. If the patient has increasing subcutaneous emphysema on exam or on repeat CXRs, the tube is left in place, and the suction is increased.

If patients had a pneumothorax on the CXR, we observe the patient if clinically stable, irrespective of the size of the pneumothorax. We repeat the CXR only in patients who have new or increasing subcutaneous emphysema and/or if they have decreasing oxygen saturation. Those who did not meet the same four criteria for chest tube removal described above on the morning of POD 1 were discharged home by 8 a.m. with the chest tube in place attached to the digital drainage system as we have previously described (3, 9).

Major and minor adverse events and readmissions within 60 days of the operation were included in the perioperative complications data. Minor and major complications were defined as Grade 1–2 and Grade 3 or higher, respectively, on the Clavien–Dindo classification system.

### Pleural space re-intervention postoperatively

Indications for reinsertion of chest tube were as follows: if the patient had symptomatic shortness of breath with reduction of their oxygen saturation on pulse oximetry and/or an increasing pneumothorax and/or increasing subcutaneous emphysema. Our criteria to perform a postoperative thoracentesis for patients after discharge were as follows: (1) if they had symptomatic increasing shortness of breath with reduction of their oxygen saturations on pulse oximetry and (2) a CXR that showed an increasing significant pleural effusion when compared with earlier postoperative CXR. We did not attempt to measure the size of an effusion's volume on CXRs or CT scans. The home criteria for thoracentesis used by home caring physicians were not objectively defined nor similar across the large number of various doctors our patients saw on follow-up once home. Patients were instructed at every touchpoint and stressed to text or call us prior to having any pleural space intervention and/or prior to going to any emergency room or follow-up doctor appointment.

### Statistical analysis

Descriptive analyses are used to report patients' baseline characteristics, intraoperative course, and postoperative outcomes. We divide the study's experience into four distinct time frames from 2009 to 2024. Categorical variables are reported as frequencies and percentages. Continuous non-normally distributed variables are reported as median with range. Statistical analyses are performed with IBM SPSS Statistics, version 26.0 (IBM; Armonk, NY, USA). The study was broken into four distinct time periods and analyzed in quartiles from 1 January 2009 until 31 December 2013 (Quartile 1), from 1 January 2014 until 31 December 2017 (Quartile 2), from 1 January 2018 until 31 December 2021 (Quartile 3), and from 1 January 2022 until 31 December 2024 (Quartile 4).

## Results

**Figure 1:**
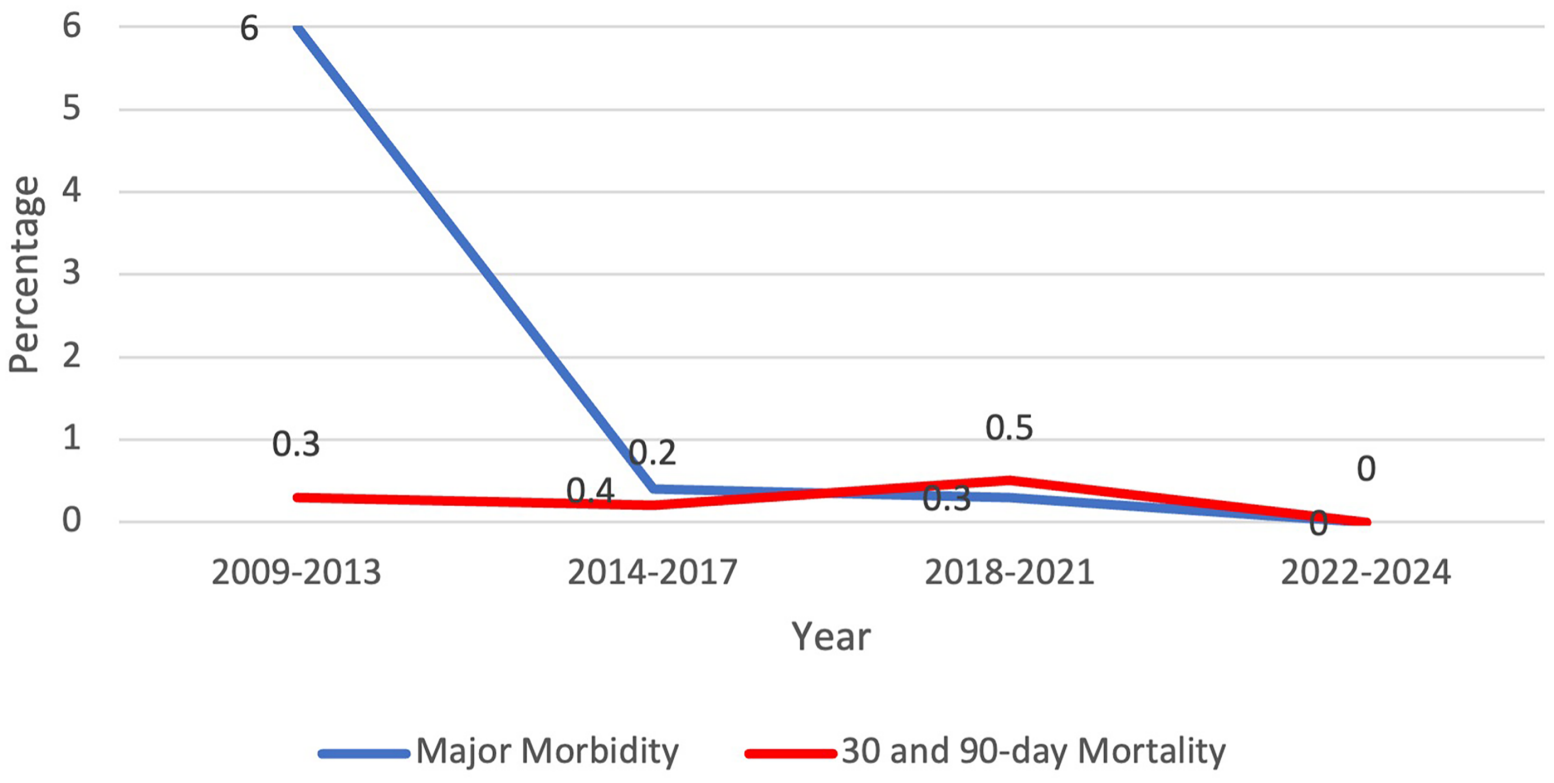
Quality metric outcome: major morbidity and 30-day and 90-day mortality decreased over the study period.

**Figure 2:**
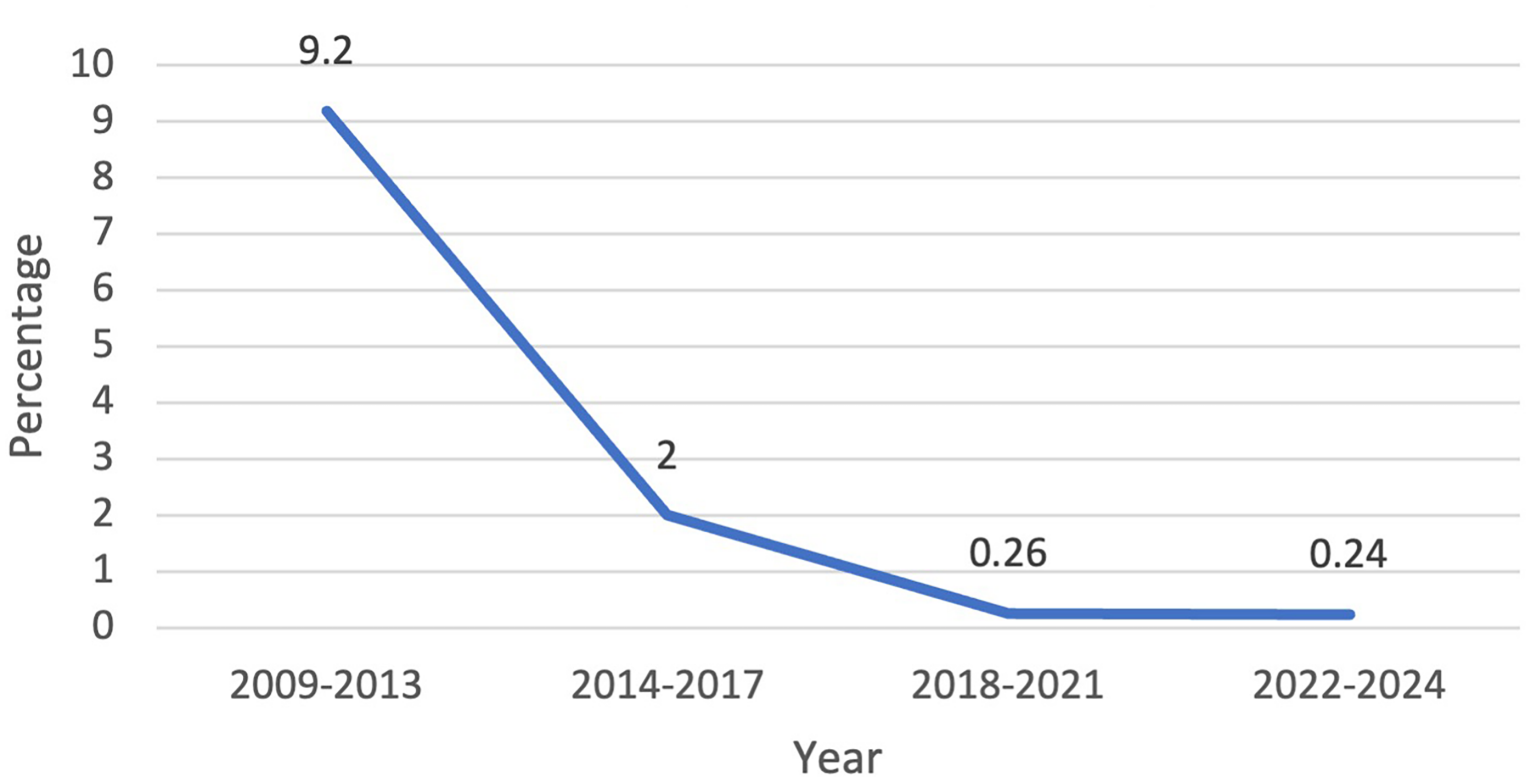
Quality metric outcome: conversions from robotic to open thoracotomy decreased from 9.2% to 0.24% over the study period.

From 1 January 2009 to 31 December 2024, there were 1,714 patients who presented to one surgeon for lobectomy or segmentectomy, and 1,701 were offered a robotic platform for resection. The other 13 patients had tumor size of 14 cm or greater. Table 1 shows the patient demographics for each quartile and overall. Table 2 depicts the operations performed. Outcomes are shown in Figure 1 (major morbidity and 30- and 90-day mortality). Major morbidity decreased from 6% to 1% (*p* < 0.001), and the 30- and 90-day mortality decreased from 0.3% to 0% (*p* < 0.001). The most common major morbidity was pneumonia, and minor morbidity was air leak and atrial fibrillation. Figure 2 shows the improved quality metric of conversions from robotic to thoracotomy from 9.1% in Quartile 1 to 0.24% in Quartile 4.

**Table 1:** Patient demographics per quartile and overall.

Variable	Quartile 1 (1/1/09–12/31/13)	Quartile 2 (1/1/14–12/31/17)	Quartile 3 (1/1/18–12/31/21)	Quartile 4 (1/1/22–12/31/24)	Total
	*N* = 409	*N* = 495	*N* = 382	*N* = 415	*N* = 1,701
Lobectomies, *n*	329	365	204	240	1,138
Segmentectomies, *n*	80	130	178	175	563
Sex, *n* (%)					
Female	192 (47%)	277 (56%)	198 (52%)	228 (55%)	895 (53%)
Male	217 (53%)	218 (44%)	184 (48%)	187 (45%)	806 (47%)
Comorbidities, *n* (%)					
Previous cancers	148 (36%)	175 (35%)	91 (24%)	101 (24%)	515 (30%)
Hypertension	319 (78%)	292 (59%)	161 (42%)	173 (42%)	945 (56%)
Diabetes	99 (24%)	102 (21%)	55 (14%)	62 (15%)	318 (19%)
Congestive heart failure	15 (4%)	41 (8%)	14 (4%)	9 (2%)	79 (5%)
Coronary artery disease	102 (25%)	98 (20%)	60 (16%)	57 (14%)	317 (19%)
Pulmonary hypertension	25 (6%)	36 (7%)	8 (2%)	18 (4%)	87 (5%)
Hyperlipidemia	184 (45%)	205 (41%)	131 (34%)	195 (47%)	715 (42%)
Chronic obstructive pulmonary disease	67 (16%)	131 (27%)	44 (12%)	42 (10%)	284 (17%)
Previous chemotherapy	56 (14%)	60 (12%)	55 (14%)	62 (15%)	233 (14%)
Previous radiation therapy	50 (12%)	35 (7%)	31 (8%)	23 (5%)	139 (8%)

**Table 2:** Types of lobectomies and segmentectomies performed and selected quality outcomes per quartile and overall.

Variable	Quartile 1 (1/1/09–12/31/13)	Quartile 2 (1/1/14–12/31/17)	Quartile 3 (1/1/18–12/31/21)	Quartile 4 (1/1/22–12/31/24)	Total
Types of lobectomies performed, *n*					
All lobectomies, *n*	329	365	204	240	1,138
Left upper lobectomy	69	54	38	44	205 (18%)
Left lower lobectomy	42	58	19	33	152 (13%)
Right upper lobectomy	143	137	77	97	454 (40%)
Right middle lobectomy	24	33	30	22	109 (10%)
Right lower lobectomy	47	73	32	38	190 (17%)
Bi-lobectomy (right sided)	4	10	8	6	28 (2%)
Types of segmentectomies performed, *n*					
All segmentectomies, *n*	80	130	178	175	563
S1	7	11	15	18	51 (9%)
S2	31	33	31	19	114 (20%)
S1 + S2	0	5	14	21	40 (7%)
S1 + S3	2	5	11	7	25 (4%)
S4 + S5	8	11	13	8	40 (7%)
S6	16	23	32	26	97 (17%)
S8	0	0	7	6	13 (2%)
S7 + S8	0	0	1	4	5 (1)
S10	0	0	5	4	9 (2%)
S6 + S10	0	0	0	6	6 (1%)
S7 + S8 + S9 + S10	1	6	0	2	9 (2%)
Others	15	36	49	54	154 (27%)
Selected quality outcomes					
Estimated blood loss in cc (median) (range)	35 (10–150)	30 (10–70)	20 (10–70)	20 (10–60)	25 (10–150)
Number of patients transfused, *n*	4	2	1	0	7
Number of patients converted from robotic to thoracotomy, *n*	30 (9.2%)	10 (2%)	1 (0.26%)	1 (0.24%)	42 (2.5%)
Metrics for patients who underwent resection for cancer					
Median number of lymph nodes resected (range)	20 (5–33)	22 (8–35)	25 (7–52)	29 (12–81)	24 (5–81)
Median number of lymph node stations assessed	5 N2	5 N2	5 N2	5 N2	5 N2
	2 N1	2 N1	3 N1	3 N1	3 N1
R0 resection	97%	98%	97%	98%	98%

**Table 3:** Press Ganey patient satisfaction scores.

Variables	Quartile 1 (1/1/09–12/31/13)	Quartile 2 (1/1/14–12/31/17)	Quartile 3 (1/1/18–12/31/21)	Quartile 4 (1/1/22–12/31/24)
Patient satisfaction score	Not available	Not available	87%	98%

**Figure 3:**
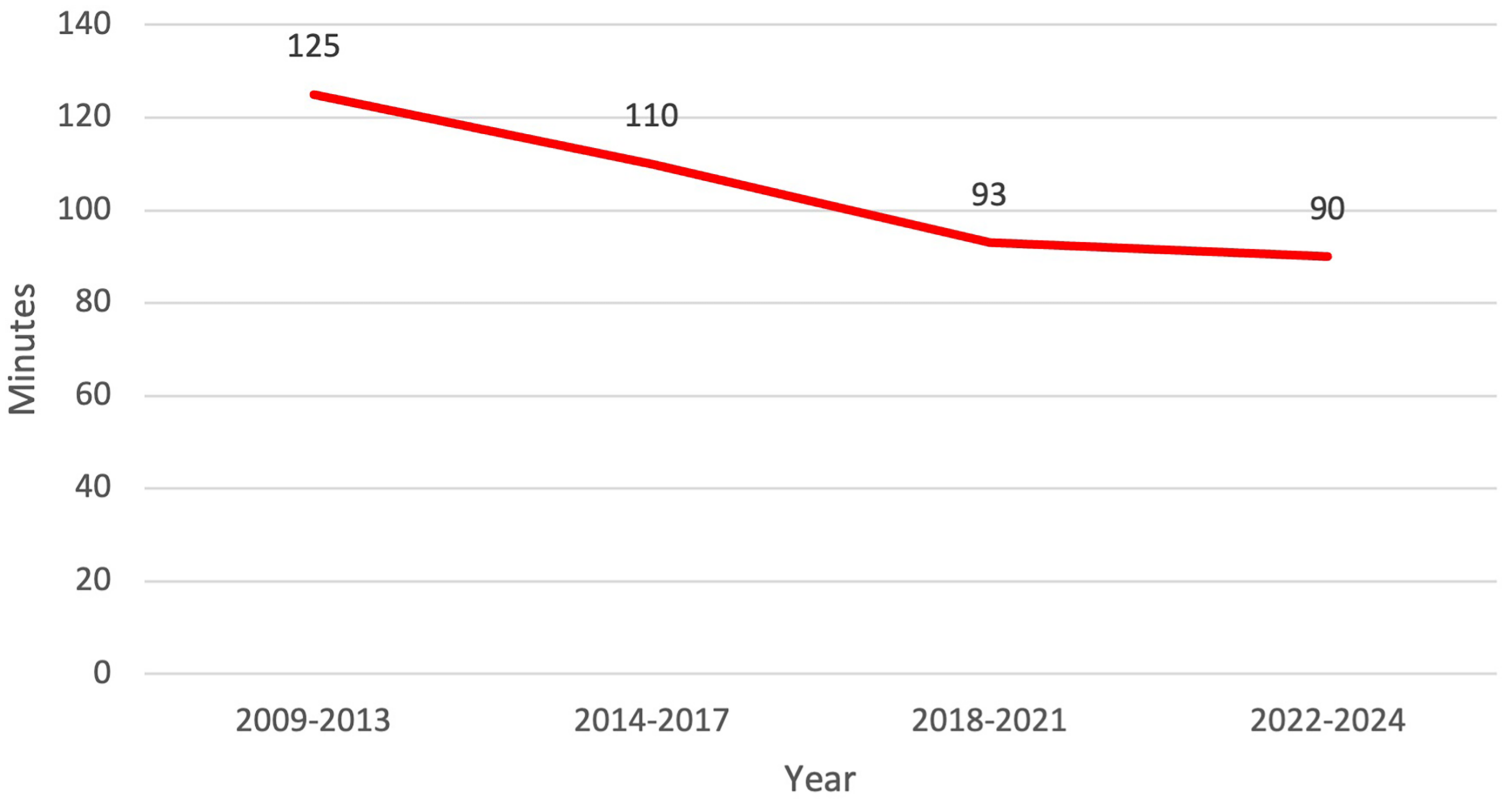
Efficiency metric outcome: decrease in operative time and total operating room dwell time from 125 to 90 min over the study period.

**Figure 4:**
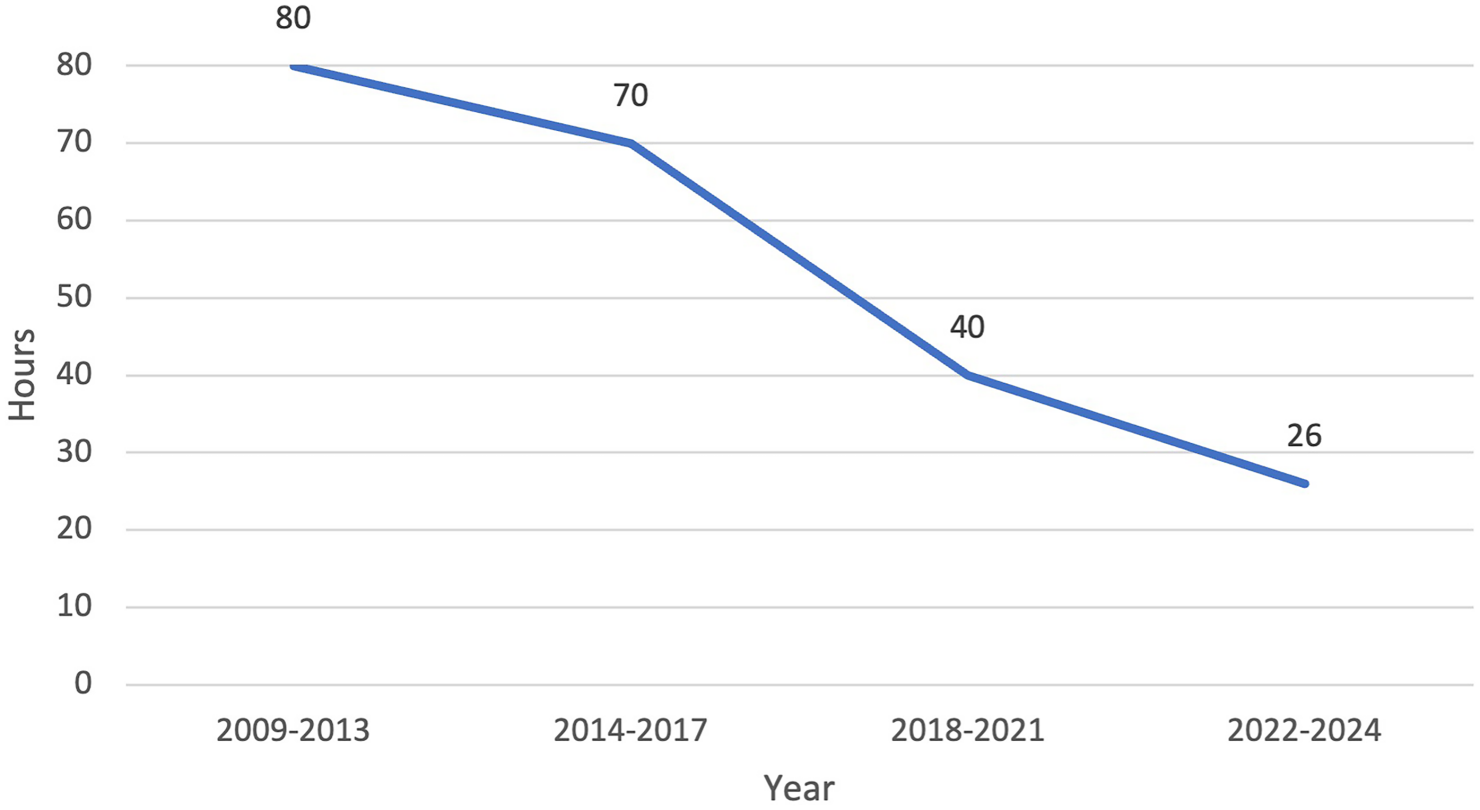
Efficiency metric outcome: decrease in median length of stay from 80 h (3.3 days) to 26 h (1 day).

**Figure 5:**
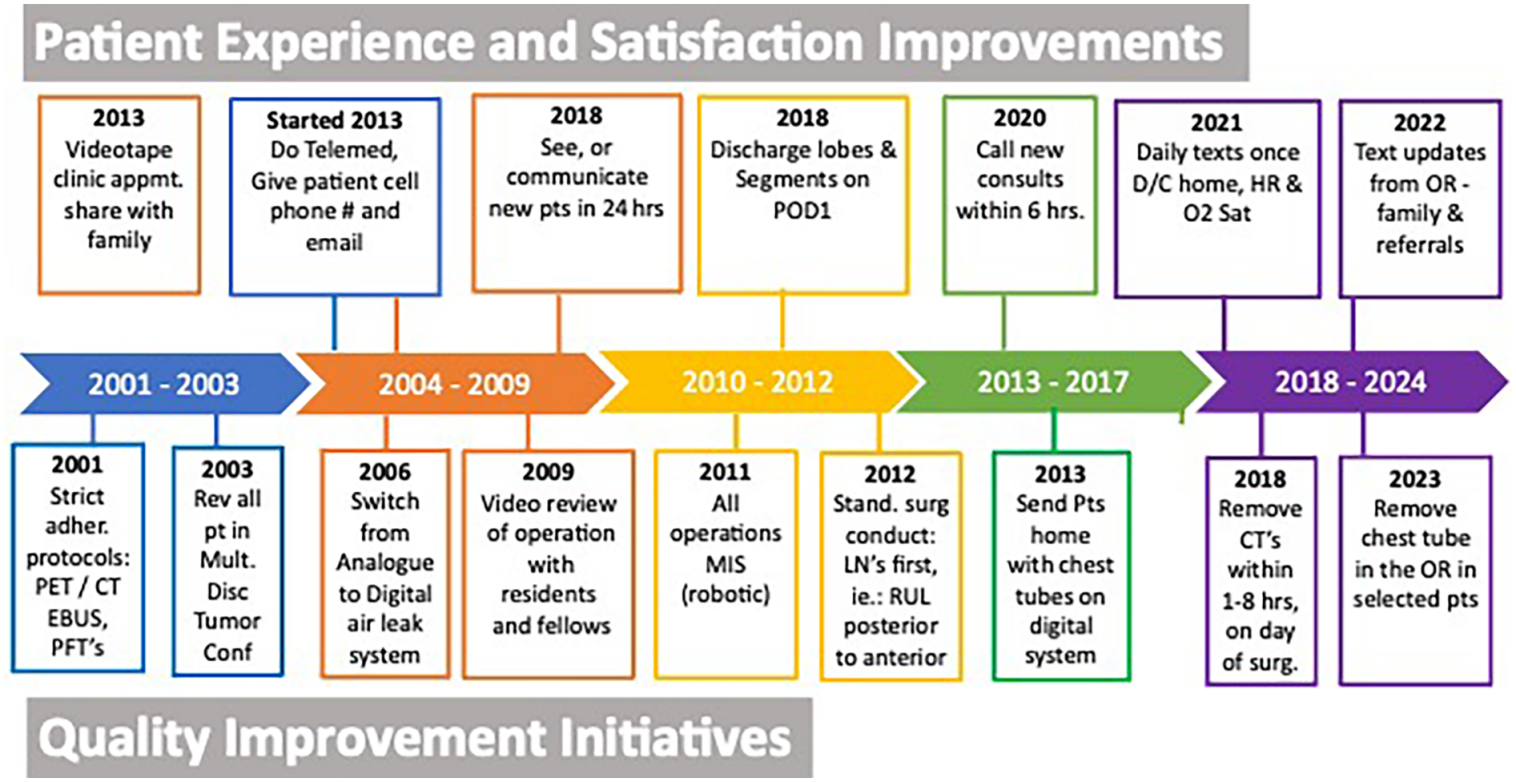
Selective process improvement changes to improve quality and efficiency outcomes and to improve patient experience and date implemented.

Our efficiency metrics improved over time as did our quality metrics (Table 3). Figure 3 depicts our median operative time which decreased from 125 min (range, 30–220 min) to 90 min (range, 29–244 min) skin-to-skin time. Figure 4 shows that the median length of stay decreased from a median of 80 h (range, 1–21 days) to 26 h (range, 1–3 days). Chest tube duration also fell from a median of 2.7 days to 6 h. In the last 6 months, it has now achieved a median of 0 h. During this time while we were sending patients home sooner, many went home with a chest tube on a digital device. Our patient satisfaction scores improved from 87% to 98% (Table 3). Figure 5 shows some of the specific process changes that we made to improve both quality outcomes and patient and family satisfaction.

## Discussion

Quality outcomes and outstanding patient experience are our ultimate goals as doctors and surgeons. Quality outcomes include many factors in our experience such as minimally invasive surgery without conversion, an R0 resection and complete thoracic lymphadenectomy for cancer patients, total operative time under 120 min, blood loss of <30 cc, length of stay in the hospital of 1 day or less, the elimination of chest tubes postoperatively either in the operating room or within 6 h postoperatively, and minimal pain and morbidity that afford quick recovery and return to all activities. Today, these quality metrics need to be accomplished while delivering the highest patient and family satisfaction and overall experience and while we still teach and train our residents and fellows (10).

Our outcomes improved over time even though we had an increasing percentage of patients with comorbidities as shown from Quartiles 1–4. This is probably related to our increasing experience in performing formal anatomic segmentectomies, especially in the basilar segments of the lower lobes and with the increasing frequency of referrals for segmentectomy and of patients with ground glass opacities. Our outcomes compare very favorably to the other largest reported robotic and video-assisted lobectomy and segmentectomy series as shown in Table 4. The major thrust of writing this paper was to evaluate our processes for us to improve and then to share some of the many painful lessons we have learned in the hope that they can be scaled and implemented by others.

**Table 4:** Comparison of our outcomes to previously reported series.

Author	Year	Database	Patients (*n*)	Operative time (minute) (median)	Number of lymph node stations	Median total lymph nodes
Cerfolio	2024	Single surgeon validated prospective	1,701	104	5 N2 3 N1	24
Reddy et al. (15)	2015	Premier Healthcare Database	838	247 (mean)	–	–
Oh et al. (16)	2015	Premier Healthcare Database	2,775	275	–	–
Kent et al. (17)	2010	State Inpatient Databases	430	–	–	–
Casiraghi et al. (18)	2016	Single institution retrospective review	339	187	3 N2 2 N1	15
Hennon et al. (19)	2014	National Cancer Database	5,470	–	–	10.9 (mean)
Cerfolio et al. (20)	2018	Multi-institution retrospective review	1,339	136	5 N2 1 N1	13

Author	R0 resection	EBL (cc) (median)	LOS (hours) (median)	Major morbidity	90-day mortality
Cerfolio	99%	35 cc	54 h	1.5%	0.2%
Reddy et al. (15)	–	–	120	33.4% (complications)	1.3% (30-day)
Oh et al. (16)	–	–	120	37.8% (complications)	1.3% (30-day)
Kent et al. (17)	–	–	96	44% (any complication)	0.2%
Casiraghi et al. (18)	–	–	120	2.4% (major complication)	0.3%
Hennon et al. (19)		–	96	–	3.2%
Cerfolio et al. (20)		50	72	8%	0.5%

Figure 5 is perhaps the most important information shared and at the same time the least scientific. We cannot prove that the specific steps or processes shown in that figure were the direct cause of the improved quality and patient experience. In addition, we cannot eliminate any of the other confounding variables or biases, such as our increasing experience, and we are unable to report all the process changes we implemented that may have contributed to our improved outcomes. Time and experience are inherent variables. In general, we all become better at patient selection, at surgery, and at leading. This study does not, nor cannot, control for this variable as well. We believe improved leadership drives each team member to perform better and all of these factors improve outcomes.

Our operative technique has advanced over time with experience which has contributed to an improvement in outcomes such as decreased major morbidity and decreased rate of conversion to thoracotomy. We believe the main driver of this improvement is a video review of our robotic operations that studies every movement and continuously refines and standardizes our operations. For instance, we now routinely perform right upper lobe resections from a posterior to an anterior approach. We believe this is safe and leads to few intraoperative complications and less air leaks because the bronchus is divided before the A1 and A3 trunk and before the superior pulmonary vein. Second, we also routinely remove all lymph nodes first and then pack those stations (2R, 4R, and 7) and later examine the nodal basin after the lung resection to allow time for subtle chyle leaks to appear. This has eliminated the low-volume chylothoraces we used to have (11), especially in the 2R location. Third, we routinely employ several strategies in patients that have difficult pulmonary artery dissection either from induction chemo/immunotherapy and/or radiotherapy. We now commonly divide the intended resected airway (bronchus) first and then control the pulmonary arterial branches later. Fourth, we have become more comfortable with performing pulmonary artery sleeve resections as well as controlling the main pulmonary artery intrapericardially on the left side or under the superior vena cava on the right side which have mitigated and almost eliminated conversions.

We believe morbidity has decreased not only because of our improvement in technique, but because we have performed the operations more quickly over time. Shorter operations reduce total anesthetic time which decreases postoperative morbidity. Since we work at an academic institution, we have been able to shorten operative time while still ensuring that residents participate fully in the operation and have not compromised their education. We have done this by ensuring the house staff is fully trained on the simulator before using the console in real time and by dividing the operation into defined, reproducible parts, as previously published (12). Perhaps most importantly, we ensure the attending surgeon's presence for the entirety of the operation from the patient's entry into the operating room to skin closure.

Another significant factor that has led to reduced morbidity is removing chest tubes sooner. This has been accomplished in part by softer and smarter lung retraction that avoids air leaks that are remote from the stapling sites. Previously, from January 2022 to August 2023, we removed chest tubes within 4–12 h after ingesting ice cream in the recovery room (6, 7). Most recently, this has allowed us to innovate a new “chest-tube-less approach” where we now try to remove the chest tube prior to the patient leaving the operating room. The digital air leak system also helps us to remove the tubes sooner, as described previously (13, 14). In addition, we have recently reduced patient pain by trying a new “chest-incision-less approach” which features all of the robotic ports and incisions placed inferior to all of the intercostal nerves (multiarm, robotic percutaneous subcostal technique).

Getting patients home sooner also improves outcomes. It prevents nosocomial infections and confusion, improves sleep hygiene, reduces medication errors, and improves patient and family satisfaction. Patients walk more at home and eat and sleep better. Once home, we ask for daily communication from the patient and/or family to the attending surgeon via text messaging of the patient's pulse oximetry data (oxygen saturation and heart rate) twice a day for 3–5 days which affords an early-warning signal if the patient is having issues such as atrial fibrillation, shortness of breath, and/or respiratory demise. In our experience, early detection and thus intervention significantly impact care and outcomes. Leveraging technology (texts, video calls, and accurate pulse oximetry data) serves as a critical early-warning system and reduces costs while improving patient care. It also prevents unnecessary visits to the emergency room which reduces patient experience and satisfaction and leads to unnecessary admissions.

Figure 5 shows some of the innovations we have made to improve patient experience. These changes, like the ones described for the quality process changes above, cannot be proven with a *p*-value, nor can they be proven to be the direct or sole cause of the increased patient satisfaction. We try to innovate quarterly and develop novel ideas that are simple and easily scalable, require little to no infrastructure, and are inexpensive. Some of the most impactful ideas are seeing any new consult by telemedicine (or phone) within 6–12 h of office contact, walking patients into the operating room, and texting the family and patient several times during the operation from the robotic console and at least once a day when patients are home for 3–5 days postoperatively.

This study has several limitations including the inability to exclude the increasing surgeons' experience and improved patient selection and to control for other unknown or unmeasurable variables that are inherently intertwined with time. The strength of this study is it is a non-selective, consecutive series where all (except for 13 patients with tumor size 14 cm or greater) were offered and underwent anatomic robotic lung resection and it uses a prospective validated database over a long period of time.

In conclusion, we believe that a commitment to continued innovation and process improvement of the entire care plan of patients who undergo robotic anatomic pulmonary resection leads to improved outcomes for our patients and improved patient and family experience. The mindset that every part of our protocols can and should get better is critical. We believe that many of the processes shown in this study can be implemented by others and are scalable. There remain many opportunities to further improve so our patients can receive higher quality care.

## Data Availability

The raw data supporting the conclusions of this article will be made available by the authors, without undue reservation.

## References

[1] Cerfolio RJ Steenwyk BL Watson C Sparrow J Belopolsky V Townsley M Decreasing the preincision time for pulmonary lobectomy: the process of lean and value stream mapping. Ann Thorac Surg. (2016) 101(3):1110–5. 10.1016/j.athoracsur.2015.09.004 26602005

[2] Cerfolio RJ Minnich DJ Wei B Watson C DeCamp MM. Achieving a 3-star Society of Thoracic Surgery lobectomy ranking by using continuing process improvement, lean methodology, and root cause analysis. Semin Thorac Cardiovasc Surg. (2017) 29(4):550–7. 10.1053/j.semtcvs.2017.08.004 28982549

[3] Geraci TC Chang SH Chen S Ferrari-Light D Cerfolio RJ. Discharging patients by postoperative day one after robotic anatomic pulmonary resection. Ann Thorac Surg. (2022) 114(1):234–40. 10.1016/j.athoracsur.2021.06.088 34389302

[4] Bryant AS Cerfolio RJ. The influence of preoperative risk stratification on fast-tracking patients after pulmonary resection. Thorac Surg Clin. (2008) 18(1):113–8. 10.1016/j.thorsurg.2007.10.002 18402207

[5] Ramadan OI Wei B Cerfolio RJ. Robotic surgery for lung resections-total port approach: advantages and disadvantages. J Vis Surg. (2017) 3:22. 10.21037/jovs.2017.01.06 29078585 PMC5637951

[6] Cerfolio RJ McCormack AJ. Innovation: ice cream in the recovery room rules out chylothorax after thoracic lymphadenectomy and affords same-day chest tube removal. Front Surg. (2024) 11:1457561. 10.3389/fsurg.2024.1457561 39193401 PMC11347312

[7] McCormack AJ El Zaeedi M Geraci TC Cerfolio RJ. The process and safety of removing chest tubes 4 to 12 h after robotic pulmonary lobectomy and segmentectomy. JTCVS Open. (2023) 16:909–15. 10.1016/j.xjon.2023.09.028 38204643 PMC10775092

[8] Cerfolio RJ Minnich DJ Bryant AS. The removal of chest tubes despite an air leak or a pneumothorax. Ann Thorac Surg. (2009) 87(6):1690–4; discussion 1694–6. 10.1016/j.athoracsur.2009.01.077 19463579

[9] Geraci TC McCormack AJ Cerfolio RJ. Discharging patients home with a chest tube and digital system after robotic lung resection. Ann Thorac Surg. (2024) 118(4):811–6. 10.1016/j.athoracsur.2024.05.004 38789008

[10] Cerfolio RJ Chang SH. Efficiency quality index (EQI)-implementing a novel metric that delivers overall institutional excellence and value for patients. Front Surg. (2020) 7:604916. 10.3389/fsurg.2020.604916 33598477 PMC7882676

[11] Bryant AS Minnich DJ Wei B Cerfolio RJ. The incidence and management of postoperative chylothorax after pulmonary resection and thoracic mediastinal lymph node dissection. Ann Thorac Surg. (2014) 98(1):232–5; discussion 235–7. 10.1016/j.athoracsur.2014.03.003 24811982

[12] Cerfolio RJ Cichos KH Wei B Minnich DJ. Robotic lobectomy can be taught while maintaining quality patient outcomes. J Thorac Cardiovasc Surg. (2016) 152(4):991–7. 10.1016/j.jtcvs.2016.04.085 27292875

[13] Cerfolio RJ Bryant AS. The benefits of continuous and digital air leak assessment after elective pulmonary resection: a prospective study. Ann Thorac Surg. (2008) 86(2):396–401. 10.1016/j.athoracsur.2008.04.016 18640304

[14] Geraci TC Sorensen A James L Chen S El Zaeedi M Cerfolio RJ Use of a novel digital drainage system after pulmonary resection. J Thorac Dis. (2022) 14(9):3145–53. 10.21037/jtd-22-574 36245636 PMC9562523

[15] Reddy RM Gorrepati ML Oh DS Mehendale S Reed MF. Robotic-assisted versus thoracoscopic lobectomy outcomes from high-volume thoracic surgeons. Ann Thorac Surg. (2018) 106(3):902–8. 10.1016/j.athoracsur.2018.03.048 29704479

[16] Oh DS Reddy RM Gorrepati ML Mehendale S Reed MF. Robotic-assisted, video-assisted thoracoscopic and open lobectomy: propensity-matched analysis of recent premier data. Ann Thorac Surg. (2017) 104(5):1733–40. 10.1016/j.athoracsur.2017.06.020 29054214

[17] Kent M Wang T Whyte R Curran T Flores R Gangadharan S. Open, video-assisted thoracic surgery, and robotic lobectomy: review of a national database. Ann Thorac Surg. (2014) 97(1):236–42. 10.1016/j.athoracsur.2013.07.117 24090577

[18] Casiraghi M Galetta D Borri A Tessitore A Romano R Diotti C Ten years’ experience in robotic-assisted thoracic surgery for early stage lung cancer. Thorac Cardiovasc Surg. (2019) 67(7):564–72. 10.1055/s-0038-1639575 29605962

[19] Hennon MW DeGraaff LH Groman A Demmy TL Yendamuri S. The association of nodal upstaging with surgical approach and its impact on long-term survival after resection of non-small-cell lung cancer. Eur J Cardiothorac Surg. (2020) 57(5):888–95. 10.1093/ejcts/ezz320 31764992 PMC7179045

[20] Cerfolio RJ Ghanim AF Dylewski M Veronesi G Spaggiari L Park BJ. The long-term survival of robotic lobectomy for non-small cell lung cancer: a multi-institutional study. J Thorac Cardiovasc Surg. (2018) 155(2):778–86. 10.1016/j.jtcvs.2017.09.016 29031947 PMC5896345

